# Genetic patterns related to von Willebrand factor: implications on the need for mechanical ventilation, severity, and death in COVID-19

**DOI:** 10.3389/fmed.2025.1690764

**Published:** 2026-01-09

**Authors:** José López Castro, Silvia Diz-de Almeida, Manuel L. López Reboiro, Cristina Sardiña González, SCOURGE Cohort Group, Javier Abellan, René Acosta-Isaac, Jose María Aguado, Carlos Aguilar, Sergio Aguilera-Albesa, Abdolah Ahmadi Sabbagh, Jorge Alba, Sergiu Albu, Karla A.M. Alcalá-Gallardo, Julia Alcoba-Florez, Sergio Alcolea Batres, Holmes Rafael Algarin-Lara, Virginia Almadana, Julia Almeida, Berta Almoguera, María R. Alonso, Nuria Alvarez, Rodolfo Alvarez-Sala Walther, Álvaro Andreu-Bernabeu, Maria Rosa Antonijoan, Eunate Arana-Arri, Carlos Aranda, Celso Arango, Carolina Araque, Nathalia K. Araujo, Izabel M.T. Araujo, Ana C. Arcanjo, Ana Arnaiz, Francisco Arnalich Fernández, María J. Arranz, José Ramon Arribas Lopez, Maria-Jesus Artiga, Yubelly Avello-Malaver, Carmen Ayuso, Ana Margarita Baldión, Belén Ballina Martín, Raúl C. Baptista-Rosas, Andrea Barranco-Díaz, María Barreda- Sánchez, Viviana Barrera-Penagos, Moncef Belhassen-Garcia, Enrique Bernal, David Bernal-Bello, Joao F. Bezerra, Marcos A.C. Bezerra, Natalia Blanca-López, Rafael Blancas, Lucía Boix-Palop, Alberto Borobia, Elsa Bravo, María Brion, Óscar Brochado-Kith, Ramón Brugada, Matilde Bustos, Alfonso Cabello, Juan J. Caceres-Agra, Esther Calbo, Enrique J. Calderón, Shirley Camacho, Marcela C. Campos, Cristina Carbonell, Servando Cardona-Huerta, Antonio Augusto F. Carioca, Maria Sanchez Carpintero, Carlos Carpio Segura, Thássia M.T. Carratto, José Antonio Carrillo-Avila, Maria C.C. Carvalho, Carlos Casasnovas, Luis Castano, Carlos F. Castaño, Jose E. Castelao, Aranzazu Castellano Candalija, María A. Castillo, Yolanda Cañadas, Francisco C. Ceballos, Jessica G. Chaux, Walter G. Chaves- Santiago, Sylena Chiquillo-Gómez, Marco A. Cid-Lopez, Oscar Cienfuegos-Jimenez, Rosa Conde-Vicente, M. Lourdes Cordero-Lorenzana, Dolores Corella, Almudena Corrales, Jose L. Cortes-Sanchez, Marta Corton, Tatiana X. Costa, Raquel Cruz, Marina S. Cruz, Luisa Cuesta, Gabriela C.R. Cunha, David Dalmau, Raquel C.S. Dantas-Komatsu, M. Teresa Darnaude, Alba De Martino-Rodríguez, Juan De la Cruz Troca, Juan Delgado-Cuesta, Aranzazu Diaz de Bustamante, Covadonga M. Diaz-Caneja, Beatriz Dietl, Silvia Diz-de Almeida, Elena Domínguez-Garrido, Alice M. Duarte, Anderson Díaz-Pérez, Jose Echave-Sustaeta, Rocío Eiros, César O. Enciso-Olivera, Gabriela Escudero, Pedro Pablo España, Gladys Mercedes Estigarribia Sanabria, María Carmen Fariñas, Marianne R. Fernandes, Lidia Fernandez-Caballero, María J. Fernandez-Nestosa, Ramón Fernández, Silvia Fernández Ferrero, Yolanda Fernández Martínez, Ana Fernández-Cruz, Uxía Fernández-Robelo, Amanda Fernández-Rodríguez, Marta Fernández-Sampedro, Ruth Fernández-Sánchez, Tania Fernández-Villa, Carmen Fernéndez Capitán, Patricia Flores-Pérez, Vicente Friaza, Lácides Fuenmayor-Hernández, Marta Fuertes Núñez, Victoria Fumadó, Ignacio Gadea, Lidia Gagliardi, Manuela Gago-Domínguez, Natalia Gallego, Cristina Galoppo, Carlos Garcia-Cerrada, Josefina Garcia-García, Inés García, Mercedes García, Leticia García, María Carmen García Torrejón, Irene García-García, Carmen García-Ibarbia, Andrés C. García-Montero, Ana García-Soidán, Elisa García-Vázquez, Aitor García-de-Vicuña, Emiliano Garza-Frias, Angela Gentile, Belén Gil-Fournier, Fernan Gonzalez Bernaldo de Quirós, Manuel Gonzalez-Sagrado, Hugo Gonzalo Benito, Beatriz González Álvarez, Anna González-Neira, Javier González-Peñas, Oscar Gorgojo-Galindo, Florencia Guaragna, Genilson P. Guegel, Beatriz Guillen-Guio, Encarna Guillen-Navarro, Pablo Guisado-Vasco, Luz D. Gutierrez-Castañeda, Juan F. Gutiérrez-Bautista, Luis Gómez Carrera, María Gómez García, Ángela Gómez Sacristán, Javier Gómez-Arrue, Mario Gómez-Duque, Miguel Górgolas, Sarah Heili-Frades, Estefania Hernandez, Luis D. Hernandez-Ortega, Cristina Hernández Moro, Guillermo Hernández-Pérez, Rebeca Hernández-Vaquero, Belen Herraez, M. Teresa Herranz, María Herrera, María José Herrero, Antonio Herrero-Gonzalez, Juan P. Horcajada, Natale Imaz-Ayo, Maider Intxausti-Urrutibeaskoa, Rafael H. Jacomo, Rubén Jara, Perez Maria Jazmin, María A. Jimenez-Sousa, Ángel Jiménez, Pilar Jiménez, Ignacio Jiménez-Alfaro, Iolanda Jordan, Rocío Laguna-Goya, Daniel Laorden, María Lasa-Lazaro, María Claudia Lattig, Ailen Lauriente, Anabel Liger Borja, Lucía Llanos, Esther Lopez-Garcia, Rosario Lopez-Rodriguez, Leonardo Lorente, José E. Lozano, María Lozano-Espinosa, Andre D. Luchessi, Eduardo López Granados, Amparo López-Bernús, Miguel A. López-Ruz, Ignacio Mahillo, Esther Mancebo, Carmen Mar, Cristina Marcelo Calvo, Miguel Marcos, Alba Marcos-Delgado, Pablo Mariscal Aguilar, Marta Martin-Fernandez, Laura Martin-Pedraza, Amalia Martinez, Iciar Martinez-Lopez, Oscar Martinez-Nieto, Pedro Martinez-Paz, Angel Martinez-Perez, Michel F. Martinez-Resendez, María M. Martín, María Dolores Martín, Vicente Martín, Caridad Martín-López, José-Ángel Martín-Oterino, María Martín-Vicente, Ricardo Martínez, Juan José Martínez, Silvia Martínez, Violeta Martínez Robles, Eleno Martínez-Aquino, Óscar Martínez-González, Andrea Martínez-Ramas, Laura Marzal, Alicia Marín Candon, Juliana F. Mazzeu, Jeane F.P. Medeiros, Kelliane A. Medeiros, Francisco J. Medrano, Xose M. Meijome, Natalia Mejuto-Montero, Humberto Mendoza Charris, Eleuterio Merayo Macías, Fátima Mercadillo, Arieh R. Mercado-Sesma, Pablo Minguez, Antonio J J. Molina, Elena Molina-Roldán, Juan José Montoya, Vitor M.S. Moraes, Patricia Moreira-Escriche, Xenia Morelos-Arnedo, Victor Moreno Cuerda, Alberto Moreno Fernández, Antonio Moreno-Docón, Junior Moreno-Escalante, Rubén Morilla, Patricia Muñoz García, Ana Méndez-Echevarria, Pablo Neira, Julian Nevado, Israel Nieto-Gañán, Joana F.R. Nunes, Rocio Nuñez- Torres, Antònia Obrador-Hevia, J. Gonzalo Ocejo-Vinyals, Virginia Olivar, Silviene F. Oliveira, Lorena Ondo, Alberto Orfao, Luis Ortega, Eva Ortega-Paino, Fernando Ortiz-Flores, Rocio Ortiz-Lopez, José A. Oteo, Harry Pachajoa, Manuel Pacheco, Fredy Javier Pacheco-Miranda, Irene Padilla Conejo, Sonia Panadero-Fajardo, Mara Parellada, Roberto Pariente-Rodríguez, Estela Paz-Artal, Germán Peces-Barba, Miguel S. Pedromingo Kus, Celia Perales, Patricia Perez, Gustavo Perez-de-Nanclares, Teresa Perucho, Lisbeth A. Pichardo, Susana M.T. Pinho, Mel·lina Pinsach-Abuin, Luz Adriana Pinzón, Guillermo Pita, Francesc Pla-Junca, Laura Planas-Serra, Ericka N. Pompa-Mera, Gloria L. Porras-Hurtado, Aurora Pujol, César Pérez, Felipe Pérez-García, Patricia Pérez-Matute, Alexandra Pérez-Serra, M. Elena Pérez-Tomás, María Eugenia Quevedo Chávez, Maria Angeles Quijada, Inés Quintela, Diana Ramirez-Montaño, Soraya Ramiro León, Pedro Rascado Sedes, Delia Recalde, Emma Recio-Fernández, Salvador Resino, Adriana P. Ribeiro, Carlos S. Rivadeneira-Chamorro, Diana Roa-Agudelo, Montserrat Robelo Pardo, Marilyn Johanna Rodriguez, German Ezequiel Rodriguez Novoa, Fernando Rodriguez-Artalejo, Carlos Rodriguez-Gallego, José A. Rodriguez-Garcia, María A. Rodriguez-Hernandez, Antonio Rodriguez-Nicolas, Agustí Rodriguez-Palmero, Paula A. Rodriguez-Urrego, Belén Rodríguez Maya, Marena Rodríguez-Ferrer, Emilio Rodríguez-Ruiz, Federico Rojo, Andrea Romero-Coronado, Filomeno Rondón García, Lidia S. Rosa, Antonio Rosales-Castillo, Cladelis Rubio, María Rubio Olivera, Montserrat Ruiz, Francisco Ruiz-Cabello, Eva Ruiz-Casares, Juan J. Ruiz-Cubillan, Javier Ruiz-Hornillos, Pablo Ryan, Hector D. Salamanca, Lorena Salazar-García, Giorgina Gabriela Salgueiro Origlia, Cristina Sancho- Sainz, Anna Sangil, Arnoldo Santos, Ney P.C. Santos, Agatha Schlüter, Sonia Segovia, Alex Serra-Llovich, Fernando Sevil Puras, Marta Sevilla Porras, Miguel A. Sicolo, Vivian N. Silbiger, Nayara S. Silva, Fabiola T.C. Silva, Cristina Silván Fuentes, Jordi Solé-Violán, José Manuel Soria, Jose V. Sorlí, Renata R. Sousa, Juan Carlos Souto, Karla S.C. Souza, Vanessa S. Souza, John J. Sprockel, David A. Suarez-Zamora, José Javier Suárez-Rama, Pedro-Luis Sánchez, Antonio J. Sánchez López, María Concepción Sánchez Prados, Javier Sánchez Real, Jorge Sánchez Redondo, Clara Sánchez-Pablo, Olga Sánchez-Pernaute, Xiana Taboada-Fraga, Eduardo Tamayo, Alvaro Tamayo-Velasco, Juan Carlos Taracido-Fernandez, Nathali A.C. Tavares, Carlos Tellería, Jair Antonio Tenorio Castaño, Alejandro Teper, Ronald P. Torres Gutiérrez, Juan Torres-Macho, Lilian Torres-Tobar, Jesús Troya, Miguel Urioste, Juan Valencia-Ramos, Agustín Valido, Juan Pablo Vargas Gallo, Belén Varón, Romero H.T. Vasconcelos, Tomas Vega, Santiago Velasco-Quirce, Julia Vidán Estévez, Miriam Vieitez-Santiago, Carlos Vilches, Lavinia Villalobos, Felipe Villar, Judit Villar-Garcia, Cristina Villaverde, Pablo Villoslada-Blanco, Ana Virseda-Berdices, Valentina Vélez-Santamaría, Virginia Víctor, Zuleima Yáñez, Antonio Zapatero-Gaviria, Ruth Zarate, Sandra Zazo, Gabriela V. da Silva, Raimundo de Andrés, Jéssica N.G. de Araújo, Carmen de Juan, Julianna Lys de Sousa Alves Neri, Carmen de la Horra, Ana B. de la Hoz, Victor del Campo-Pérez, Manoella do Monte Alves, Katiusse A. dos Santos, Yady Álvarez-Benítez, Felipe Álvarez-Navia, María Íñiguez, Miguel López de Heredia, Ingrid Mendes, Rocío Moreno, Esther Sande, Carlos Flores, José A. Riancho, Augusto Rojas-Martinez, Pablo Lapunzina, Angel Carracedo, Jose A. Riancho, Augusto Rojas-Martinez, Pablo Lapunzina, Carlos Flores, Raquel Cruz, Angel Carracedo

**Affiliations:** 1 Internal Medicine Department, Hospital Público de Monforte de Lemos, Lugo, Spain; 2 Fundación Instituto de Investigación Sanitaria (FIDIS) Servicio Galego de Saúde (SERGAS), Santiago de Compostela, Spain; 3 Genomics and Bioinformatics Group, Center for Research in Molecular Medicine and Chronic Diseases (CiMUS), Universidade de Santiago de Compostela (USC), Santiago de Compostela, Spain; 4 Centro de Investigación en Red de Enfermedades Raras (CIBERER), Instituto de Salud Carlos III, Madrid, Spain; 5 IDIVAL, Santander, Spain; 6 Universidad de Cantabria, Santander, Spain; 7 Hospital U M Valdecilla, Santander, Spain; 8 Tecnologico de Monterrey, Escuela de Medicina y Ciencias de la Salud, Monterrey, Mexico; 9 Instituto de Genética Médica y Molecular (INGEMM), Hospital Universitario La Paz-IDIPAZ, Madrid, Spain; 10 ERN-ITHACA-European Reference Network, Centro de Investigación en Red de Enfermedades Raras (CIBERER), Instituto de Salud Carlos III, Madrid, Spain; 11 Research Unit, Hospital Universitario Nuestra Señora de Candelaria, Instituto de Investigación Sanitaria de Canarias, Santa Cruz de Tenerife, Spain; 12 Centro de Investigación Biomédica en Red de Enfermedades Respiratorias (CIBERES), Instituto de Salud Carlos III, Madrid, Spain; 13 Genomics Division, Instituto Tecnológico y de Energías Renovables, Santa Cruz de Tenerife, Spain; 14 Facultad de Ciencias de la Salud, Universidad Fernando Pessoa Canarias, Las Palmas de Gran Canaria, Las Palmas de Gran Canaria, Spain; 15 Fundación Pública Galega de Medicina Xenómica, Santiago de Compostela, Spain; 16 Genomics and Bioinformatics Group, Center for Research in Molecular Medicine and Chronic Diseases (CiMUS), Universidade de Santiago de Compostela (USC), Santiago de Compostela, Spain; 17 Centro de Investigación en Red de Enfermedades Raras (CIBERER), Instituto de Salud Carlos III, Madrid, Spain; 18 Instituto de Investigación Sanitaria de Santiago (IDIS), Santiago de Compostela, Spain

**Keywords:** COVID-19 severity, invasive mechanical ventilation, von Willebrand factor, VWF gene, admixed population

## Abstract

**Background:**

Elevated levels of Von Willebrand Factor (VWF) have been associated to an increased need of mechanical ventilation and higher mortality risk in COVID-19 patients, but the hypothesis of a shared genetic background has not been explored.

**Methods:**

Common and low-frequency genetic variants belonging to the *VWF*, *FVIII*, and *ADAMTS13* genes were tested for association with clinical variables related to severe COVID-19 disease in 9,371 European and 3,495 Latin-American patients. Gene–environment and gene–gene interactions were also explored.

**Results:**

A variant in the *VWF* gene was associated to the need of invasive mechanical ventilation (IMV) in the Latin-American population. Gene–gene interaction models pointed to an interaction between *ADAMTS13* and *VWF* genes.

**Conclusion:**

Although we did not find significant associations among Europeans, we identified a low-frequency variant belonging to the *VWF* gene associated with the need of IMV in Latin-Americans.

## 1 Introduction

Endothelial damage caused by SARS-CoV-2 has been documented in several recent studies and is closely related to the thrombotic complications observed in severe COVID-19 (1–3), which are associated with a higher risk of mechanical ventilation (MV) and mortality. Severe COVID-19 cases often present a procoagulant state, characterized by elevated D-dimer and thrombin levels that promote microthrombosis and chronic reactive endotheliitis. In this context, the von Willebrand Factor plays a key role in endothelial activation and platelet adhesion, and its dysregulation has been proposed as a potential biomarker for severe COVID-19 (4). Some scores already exploit the prognostic value of D-dimer and thrombin levels to evaluate the prognosis of the disease (5), and lower concentrations of VWF multimers in plasma have been correlated to the O allele in the ABO blood group (6), while the O associates with protection in SARS-CoV-2 infection (7, 8). Likewise, MV (invasive or non-invasive) and mortality have been directly associated to lupus anticoagulant levels above 1.1 IU and VWF levels>200% (3), which is another indicator of the thrombogenesis associated to SARS-CoV-2 infection (3). Taken together, the data suggest a strong thrombophilic genetic fingerprint in patients who develop severe COVID-19 disease.

Numerous international initiatives, including the HGI consortium (9) and GenOMICC (10), have worked to identify the genetic basis of COVID-19, resulting in the discovery of more than 30 variants associated with either susceptibility to infection or disease severity. However, the collaborative nature of these large-scale consortia poses difficulties for studying specific disease-related outcomes, since collecting such detailed data across participant centers is often not feasible. In this regard, the SCOURGE consortium (11) dedicated part of its efforts to gathering clinical information that could be useful for detecting genetic variation underlying different disease manifestations.

Our aim here was to study the association between critical COVID-19 disease-related outcomes and markers from the *VWF* gene. Additionally, two other genes were studied: *ADAMTS13*, a metalloprotease that cleavages VWF multimers and has been associated with heart diseases (12), and *FVIII*, since the ratio FVIII/VWF was associated with critical disease and need for MV during COVID-19 (13). These were evaluated in two independent cohorts comprising Spanish and Latin-American COVID-19 patients from the SCOURGE consortium. The inclusion of Latin-American populations in our study allows for the identification of variants that are less frequent or absent in European cohorts. The genetic composition of these populations, shaped by admixture of Native-American, European and African ancestries, translates into an increased diversity though which can improve our understanding of genetic disease risk.

## 2 Materials and methods

### 2.1 Scourge cohorts

The genetic associations with COVID-19 severity were assessed in a sample recruited between March and December 2020 for the GWAS study on COVID-19 of the Spanish Coalition to Unlock Research on Host Genetics (SCOURGE).^1^

The study comprises two cohorts: 9,371 European samples from 34 Spanish hospitals with confirmed COVID-19 diagnosis (EU cohort) and 3,495 patients from Latin-American countries and from recruitments of individuals of Latin-American descent conducted in Spain (LA cohort). Detailed data collection and quality control procedures can be found in Cruz et al. (11) and Diz-de Almeida et al. (14). Briefly, the EU sample contains COVID-19 positive cases recruited from 34 centers in 25 cities between March and December 2020 and whose estimated ancestry was >80% European. The LA cohort includes COVID-19–positive individuals recruited across five Latin American countries (Mexico, Brazil, Colombia, Paraguay, and Ecuador) between March 2020 and July 2021, and additional COVID-19–positive participants from Spain who had evidence of origin from a Latin American country or showed inferred admixture between AMR, EUR, and AFR (<0.05% SAS/EAS). We excluded individuals with more than 80% of inferred EUR ancestry as estimated by the ADMIXTURE (15) software. Genomes were imputed in the TopMed Imputation Server and only variants with imputation quality (*r*^2^) over 0.8 were kept.

The following clinical variables, all of them related to COVID-19 critical disease, were tested in our study: critical severity, defined as admission to the intensive care unit (ICU) or need of MV (invasive or non-invasive); requirement of invasive MV (IMV); pulmonary thromboembolism (PT), and hospital mortality. Controls were defined as being COVID-19 positive but not satisfying the case condition (patients classified as non-critical, not requiring IMV, not suffering from pulmonary thromboembolism or that survived).

### 2.2 Statistical analyses

Variants belonging to the *VWF*, *FVIII*, and *ADAMTS13* genes were tested for association with the above-mentioned clinical COVID-19 related variables. In this study, we did not filter the imputation results by minor allele frequency (MAF), keeping also low-frequency variants. After excluding monomorphic positions and variants with low imputation quality (*r*^2^ < 0.8), a total of 2,458, 744, and 705 genetic variants remained for the EU cohort association study of the *VWF*, *FVIII* and *ADAMTS13* genes, respectively. In the LA cohort, 3,255, 1,222, and 834 genetic variants were analyzed for *VWF*, *FVIII* and *ADAMTS13*, respectively.

Associations for each variant were assessed using logistic regression mixed models adjusted for sex, age and the top 10 genetic principal components, an approach that controls for population stratification while maximizing power (16). We tested genetic associations with critical severity, death, suffering from PT, and requirement of IMV, using the R package SAIGEgds (17). The number of cases and controls for each variable are shown in Table 1. Due to the low number of cases, the number of variants analyzed was different for each variable and cohort for some of the analyses. For this reason, the Bonferroni correction was applied in each cohort and variable, adjusting for the number total of variants analyzed that had a minor allele count (MAC) > 15, keeping a false-positive rate of 5%. Index variants were selected after LD-clumping in PLINK 1.9 (18).

**Table 1:** Number of cases (presence of phenotype) and controls (absence of phenotype) in COVID-19 patients from European and Latin-American SCOURGE cohorts.

	European cohort		Latin-American cohort	
Phenotype	N cases	N controls	N cases	N controls
Critical severity	1,125	7,771	746	2,749
Death	791	7,846	305	3,128
Pulmonary thromboembolism	241	5,483	73	2,701
Invasive mechanical ventilation	574	3,944	415	3,075

Additionally, the combined effect of several variants was tested using the multifactorial dimensionality reduction system (MDR) (19). MDR is a statistical and machine learning technique designed to identify combinations of variables (genetic or environmental factors) that interact in a nonadditive way to influence a binary outcome. A set of *n* factors is selected (by default, 1, 2, and 3), and the ratio of cases to controls is calculated for each *n* factors and their possible multi-factorial classes. Each cell is assigned to either a low- or high-risk group depending on the ratio of cases and controls. All possible factor combinations are tested sequentially, and the model with the lowest classification error between cases and controls is selected for each set of *n* factors. Model performance is assessed through a 10-fold cross-validation procedure, where 90% of the data are used for training and 10% for testing. This process is repeated 10 times using random seed numbers to minimize bias from random data partitioning.

We applied the MDR to determine the best model of 1, 2, and 3 variables for our dataset, including as independent variables gender and age (dichotomized as <60/= > 60) and a selection of good-quality and independent variants from the three genes. In each cohort, variants were filtered by MAF (>0.01), call rate (>99%) and linkage disequilibrium (pruning options in PLINK—indep-pairwise 1,000 80 0.1). Thus, in the EU cohort 138 variants were selected (38 from *FVIII*, 41 from *ADAMTS13* and 69 from *VWF*), while in the LA cohort the number of selected variants was higher, especially for the *VWF* gene (46 in *FVIII*, 42 in *ADAMTS13* and 176 in *VWF*; 264 SNPs in total). Cross-validation (CV) consistency—defined as the number of times a model is identified as the best model across the CV subsets and the average of the balance testing accuracy (the mean of sensitivity and specificity) were used to evaluate the performance of each model. We used the MDR permutation module to test the significance of the association of these final models with case status.

## 3 Results

### 3.1 Association analysis

Table 2 shows the results of logistic regression for individual genetic variants showing an association *p*-value<10^−4^ with any of the dependent variables. The full association results are provided in the Supplementary Tables 1 and 2. Results are clearly different between both cohorts. In the EU cohort, none of the variants reached significance after multi-test correction; only one variant in *VWF* was suggestively related with IMV (non-significant in the LA cohort) and other with PT (not analyzed in the LA cohort).

**Table 2:** Results of logistic regression for the top (*p* < 1×10^−3^) associated SNPs.

			European cohort					Latin-American cohort				
	SNP	Gene	AF.alt	Beta	SE	*p*-value	Bonf.	AF.alt	Beta	SE	*p*-value	Bonf.
*IMV*												
	chr12:6069602:C:T	*VWF*	0.0013	3.30	0.94	4.5E-04	2.9E-05	0.0023	−1.20	0.91	1.9E-01	2.4E-05
	chr12:6079329: A: G	*VWF*	0.0079	0.31	0.33	3.5E-01		0.0297	1.01	0.27	1.7E-04	
	chr12:6090201:C:T	*VWF*	0.0019	0.33	0.69	6.3E-01		0.0128	2.01	0.44	**3.9E-06**	
	chr12:6099270:G:A	*VWF*	0.0033	0.17	0.51	7.4E-01		0.0302	0.89	0.26	5.3E-04	
*PT*												
	chr12:6028747:G:C	*VWF*	0.0013	7.67	1.93	7.3E-05	3.3E-05	-	-	-	-	2.5E-05
*Critical*												
	chr12:6090201:C:T	*VWF*	0.0019	0.10	0.52	8.5E-01	2.9E-05	0.0127	1.32	0.34	8.0E-05	2.4E-05
	chrX:154989165:G:A	*FVIII*	0.0002	−0.69	1.17	5.6E-01		0.0069	1.22	0.33	2.3E-04	
*Death*												
	chr12:6090201:C:T	*VWF*	0.0018	0.33	0.69	6.4E-01	2.9E-05	0.0127	−1.75	0.50	4.4E-04	2.4E-05
	chrX:154989165:G: A	*FVIII*	0.0002	0.81	1.15	4.8E-01		0.0067	−2.36	0.56	*2.6E-05*	

Beta coefficients correspond to the log-odd from logistic regression models.

IMV, invasive mechanical ventilation; PT, pulmonary thromboembolism; ref, reference allele; alt, alternative allele; AF.alt, allele frequency of alternative allele; SE, standard error; Bonf, Bonferroni threshold for each analysis-cohort.

In the LA cohort, one SNP at *VWF* (chr12:6090201:C:T, rs146760599) was significantly associated with IMV (OR = 7.45 [IC: 3.15–17.68], *p* < 2.35×10^−5^, probability threshold for 2,127 markers with MAC > 15 in the LA cohort) and it also was near significance in the association with death and critical disease. Although this is a low-frequency variant, its imputation *R*^2^ was 0.96. Other two variants at *VWF* showed suggestive association with IMV in the LA cohort. All these variants showed non-significant associations in the EU cohort, where the MAF was clearly lower, in line with the recorded in gnomAD v4.1.0 (i.e., chr12:6090201:C:T European AF = 0.00028, admixed American AF = 0.009; 1 K: European AF = 0.00098, admixed American AF = 0.010). Adjustment by presence of vascular comorbidities yielded a *p*-value of 5.5×10^−6^.

Additionally, one SNP at *FVIII* (chrX:154989165:G:A, rs150171740) showed suggestive association with critical disease and death. The difference in MAF depicted in Table 2 also agrees with gnomAD population frequencies (European AF = 0.00009, admixed American AF = 0.0025). No variants within *ADAMTS13* were included in the group of top associated variants.

### 3.2 MDR results

We found some interesting results in the MDR analyses, underscoring the importance of assessing clinical risk factors and the interaction between genes. For all phenotypes the best one-variable model involved sex (for IMV and critical disease) or age (for death and PT) in both cohorts (Table 3). However, some other multiple models involving genetic variants also showed a good cross validation consistency (> = 9/10) and the highest testing balanced accuracy. A summary of these selected models can be seen in Table 3. The variant found associated with IMV (chr12:6090201:C:T, *VWF* gene, see Table 2) was also found to be involved in the best model for death in the LA-cohort (CV 10/10, 0.70 of testing accuracy). It is also interesting to note the role of variants belonging to *ADAMTS13* in two of these multivariate selected models, as this gene did not have any suggestive variants falling into the top individual association results.

**Table 3:** Results of the MDR analyses.

Cohort	Phenotype	Best Models	CV	Bal. Acc. CV testing
EU cohort	IMV	Sex	10/10	0.645
		Sex; chr9:133430501:G:A; chr12:6011265:C:T	9/10	0.647
	Death	Age	10/10	0.738
		Age; chrX:154981988	9/10	0.738
	Critical	Sex	10/10	0.650
	PT	Age	10/10	0.553
LA cohort	IMV	Age; Sex	10/10	0.620
	Death	Age	10/10	0.689
		Age; chr12:6090201:C:T	10/10	0.696
	Critical	Age; Sex	10/10	0.677
	PT	Age	10/10	0.632
		Age; chr9:133430770:C:G; chr12:5988789:G:C	9/10	0.654

Top selected models (those showing a CV > =9/10) for each dependent variable.

CV, Cross-validation consistency; is a measure of the number of times a model is identified as the best model across the CV subsets in a 10-fold cross-validation procedure. Bal. Acc. CV testing, Balance accuracy cross-validation testing is the average of the balance testing accuracy in the 10 CV subsets.

**Figure 1:**
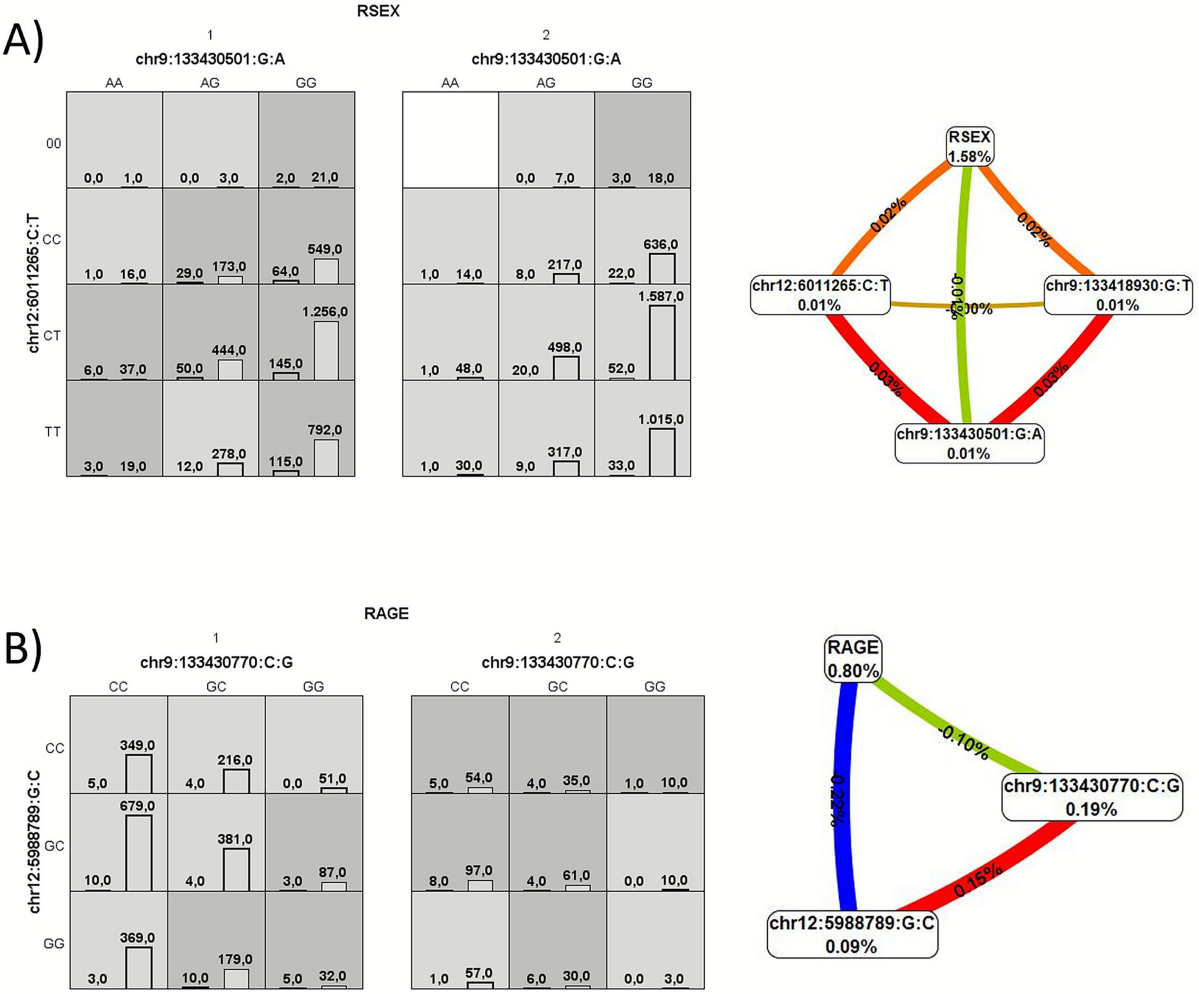
Graphical summary of MDR results for **(A)** European cohort and IMV, and **(B)** LA cohort and PT. Graphical model (left) and entropy graph (right). The graphical model (left) is a summary of variable combinations associated with high risk genotypes (dark shading) and with low risk genotypes (light shading), along with the corresponding distribution of cases (left bars in boxes) and controls (right bars in boxes). The entropy graph (right) describes the proportion of entropy that is explained by each SNP or pairwise combination within our study population. Schematic coloration used in the visualization tools represents a continuum from synergy (red or orange for strong and moderate) to redundancy (green and blue), whereas positive and negative values correspond to positive and negative interaction, respectively.

Figure 1 illustrates the graphical model (left) and the entropy graph (right) for the most interesting models in the EU and LA cohorts, respectively. Both models were significant in the permutation analysis (*p* < 0.01) and, in both cases, a synergist interaction was found between two variants, one from *VWF* and another from *ADAMTS13*. These interactions were confirmed by including the selected variables and their interactions in a logistic regression model. The interaction between chr9:133430770:C:G and chr12:5988789:G:C showed a beta coefficient of 0.8159 (*p*-value = 0.00132) in the analysis of PT in LA cohort and the interaction between chr9:133430501:G:A and chr12:6011265:C:T showed a beta coefficient of 0.3956 (*p*-value = 0.0023) in the analysis of IMV in EU cohort.

## 4 Discussion

From a clinical point of view, there is no doubt about the high thrombogenicity of SARS-CoV-2 due to endothelial dysfunction (7), causing elevated levels of prothrombotic substances such as D-dimer or VWF in critical COVID-19 patients. Some studies provided evidence of the relationship between higher levels of VWF or Lupus Anticoagulant and a higher risk of fatal events in these patients (20). Here, we aimed to evaluate whether this relationship had a genetic link. Since these biomarkers were not measured in our COVID-19 cohort, an indirect approach was implemented using several clinical variables related to COVID-19 severity. Briefly, we performed single variant (including low-frequency) analyses for the *VWF*, *ADAMTS13*, and *FVIII* genes, and subsequently conducted interaction analyses between them and with relevant clinical factors.

Although we did not find significant associations in the EU cohort, we identified a low-frequency variant belonging to the *VWF* gene (rs146760599) associated with the need of MV in the LA cohort. This variant shows marked differences in allele frequency across populations, occurring in less than 0.1% of Europeans but in 1% of admixed American individuals. Admixed populations result from the combination of multiple ancestral sources, which can lead to higher frequencies of variants that are rare or absent in other groups. Studying such populations is important for discovering genetic risk factors that may remain undetected in homogeneous cohorts. In fact, the higher frequency of this variant seems to be driven by the African local ancestry component (LA-AF_afr_ = 0.085 vs. LA-AF_eur_ = 0.0015; gnomAD v4.1.0), being much harder to detect in our European cohort even with larger sample size. This intron variant moderately correlates (*R*^2^ = 0.57) with a missense variant, rs141087261 (p.Gly967Val), which was previously reported as a frequent pathogenic variant for type 3 von Willebrand Disease in individuals with African ancestry, although it has since been classified as likely benign in Clingen. The mentioned variant was also included in the best MDR model for death due to COVID-19.

It has been established that the SARS-CoV-2 induces chronic oxidative stress at the endothelial level, causing the release of von Willebrand factor multimers and hypercoagulability (7). The von Willebrand factor then becomes an attractive independent prognostic marker of severe COVID-19 and acute respiratory distress syndrome (21). Rare variation in the *VWF* locus may thus be a relevant subject of research toward deciphering host genetic factors contributing to endothelial damage in COVID-19 patients.

No other individual associations were found within the *ADAMTS13* nor the *FVIII* genes. However, MDR analyses revealed interactions between variants belonging to different genes for need of IMV and PT (yet, the goal of MDR analyses is purely hypothesis generation). Gene–gene interactions can result in variants that have no impact by themselves on a trait but rather increase risk when present together. In this sense, the relationship between *ADAMTS13* and *VWF* is well-known (22). Research has found that critical COVID-19 patients had a lower ratio of ADAMTS13/VWF activity (23, 24), suggesting a potential role of both factors in disease progression. Other studies have shown that the dysregulation of ADAMTS13 and VWF levels is involved in diseases such as chronic thromboembolic pulmonary hypertension (25) and venous thromboembolism (26). Imbalances in the levels of both genes were found in critical patients, suggesting an altered expression. Although we could not establish a direct link between them and COVID-19 severity, our analyses pointed to a potential synergistic gene–gene interaction for outcomes such as pulmonary thromboembolism.

We would also like to acknowledge and address some of the limitations of this study. Replication was not possible because the available COVID-19 studies in AMR populations did not report results for the same variables analyzed here. In addition, the rs146760599 variant was not included in the HGI AMR A2 meta-analysis (corresponding to critical COVID-19). The absence of association in our European SCOURGE sample might be explained by differences in allele frequency or linkage-desequilibrum structure between populations, such that the causal variant could be tagged by different SNPs in other groups, and our EUR GWAS might not have been sufficiently powered to detect them. Given that this is a low-frequency variant, our findings require further validation in external cohorts with sufficient power to detect rare genetic variation. In this study, we did not directly assess the relationship between coagulation factors and COVID-19 severity, as these biochemical measurements were not available in our cohort. Hence, our results neither support nor provide a causal explanation for the genetic mechanisms underlying dysregulated levels of VWF, FVIII or ADAMTS13 in COVID-19 severe patients. Instead, they should be considered exploratory, emphasizing the need for further investigation into the role of low-frequency and rare variation in the *VWF* and *ADAMTS13* genes in critical COVID-19.

## 5 Conclusion

In the present study we have found a significant association between a low-frequency variant located in the *VWF* gene and severe COVID-19 disease in Latin-American populations, as well as exploratory gene–gene interactions between *VWF* and *ADAMTS13*. Further studies on rare variation at these loci are needed toward deciphering their contribution to endothelial damage in COVID-19.

## Data Availability

The data analyzed in this study is subject to the following licenses/restrictions: The datasets used and/or analysed during the current study are available from the corresponding author on reasonable request. Summary statistics from the SCOURGE European and Latin American GWAS and the analysis scripts are available from the public repository https://github.com/CIBERER/Scourge-COVID19 (copy archived at CIBERER, 2024). Requests to access these datasets should be directed to https://redcap.ciberisciii.es/surveys/?s=CMHFLDHXPALX3AAL.

## Footnotes

1
https://www.scourge-covid.org

## Group members of SCOURGE cohort group

SCOURGE Cohort Group members and affiliations are listed in Supplementary Material (Supplementary Table 3).
