# Supplementary material for: Genetic patterns related to von Willebrand factor: implications on the need for mechanical ventilation, severity, and death in COVID-19
Source: Front Med (Lausanne). 2026 Jan 9;12:1690764. doi: 10.3389/fmed.2025.1690764 (PMC12828584; doi:10.3389/fmed.2025.1690764)
Supplement: Supplementary file 2 [file Data_Sheet_2.docx]

Supplementary Material

**Full list of cohort members and affiliations**

## Scourge Cohort Group

Javier Abellan^1,2^; René Acosta-Isaac^3^; Jose María Aguado^4,5,6,7^; Carlos Aguilar^8^; Sergio Aguilera-Albesa^9,10^; Abdolah Ahmadi Sabbagh^11^; Jorge Alba^12^; Sergiu Albu^13,14,15^; Karla A.M. Alcalá-Gallardo^16^; Julia Alcoba-Florez^17^; Sergio Alcolea Batres^18^; Holmes Rafael Algarin-Lara^19,20^; Virginia Almadana^21^; Julia Almeida^22,23,24,25^; Berta Almoguera^26,27^; María R. Alonso^28^; Nuria Alvarez^28^; Rodolfo Alvarez-Sala Walther^18^; Álvaro Andreu-Bernabeu^29,6^; Maria Rosa Antonijoan^30^; Eunate Arana-Arri^31,32^; Carlos Aranda^33,34^; Celso Arango^29,35,6^; Carolina Araque^36,37^; Nathalia K. Araujo^38^; Izabel M.T. Araujo^39^; Ana C. Arcanjo^40,41,42^; Ana Arnaiz^43,44,45^; Francisco Arnalich Fernández^46^; María J. Arranz^47^; José Ramon Arribas Lopez^46^; Maria-Jesus Artiga^48^; Yubelly Avello-Malaver^49^; Carmen Ayuso^26,27^; Ana Margarita Baldión^49^; Belén Ballina Martín^11^; Raúl C. Baptista-Rosas^50,51,52^; Andrea Barranco-Díaz^20^; María Barreda- Sánchez^53,54^; Viviana Barrera-Penagos^49^; Moncef Belhassen-Garcia^55,56^; Enrique Bernal^53^; David Bernal-Bello^57^; Joao F. Bezerra^58^; Marcos A.C. Bezerra^59^; Natalia Blanca-López^60^; Rafael Blancas^61^; Lucía Boix-Palop^62^; Alberto Borobia^63^; Elsa Bravo^64^; María Brion^65,66^; Óscar Brochado-Kith^67,7^; Ramón Brugada^68,69,66,70^; Matilde Bustos^71^; Alfonso Cabello^72^; Juan J. Caceres-Agra^73^; Esther Calbo^74^; Enrique J. Calderón^75,76,77^; Shirley Camacho^78^; Marcela C. Campos^40^; Cristina Carbonell^79,56^; Servando Cardona-Huerta^80^; Antonio Augusto F. Carioca^81^; Maria Sanchez Carpintero^33,34^; Carlos Carpio Segura^18^; Thássia M.T. Carratto^82^; José Antonio Carrillo-Avila^83^; Maria C.C. Carvalho^84^; Carlos Casasnovas^85,86,27^; Luis Castano^31,32,27,87,88^; Carlos F. Castaño^33,34^; Jose E. Castelao^89^; Aranzazu Castellano Candalija^90^; María A. Castillo^78^; Yolanda Cañadas^34^; Francisco C. Ceballos^67^; Jessica G. Chaux^37^; Walter G. Chaves- Santiago^91,37^; Sylena Chiquillo-Gómez^19,20^; Marco A. Cid-Lopez^16^; Oscar Cienfuegos-Jimenez^80^; Rosa Conde-Vicente^92^; M. Lourdes Cordero-Lorenzana^93^; Dolores Corella^94,95^; Almudena Corrales^96,97^; Jose L. Cortes-Sanchez^80,98^; Marta Corton^26,27^; Tatiana X. Costa^99^; Raquel Cruz^100,27,101,102^; Marina S. Cruz^38^; Luisa Cuesta^103^; Gabriela C.R. Cunha^104^; David Dalmau^105,74^; Raquel C.S. Dantas-Komatsu^38^; M. Teresa Darnaude^106^; Alba De Martino-Rodríguez^107,108^; Juan De la Cruz Troca^109,110,76^; Juan Delgado-Cuesta^111^; Aranzazu Diaz de Bustamante^106^; Covadonga M. Diaz-Caneja^29,35,6^; Beatriz Dietl^74^; Silvia Diz-de Almeida^27,102^; Elena Domínguez-Garrido^112^; Alice M. Duarte^39^; Anderson Díaz-Pérez^20^; Jose Echave-Sustaeta^113^; Rocío Eiros^114^; César O. Enciso-Olivera^36,37^; Gabriela Escudero^115^; Pedro Pablo España^116^; Gladys Mercedes Estigarribia Sanabria^117^; María Carmen Fariñas^43,44,45^; Marianne R. Fernandes^118,119^; Lidia Fernandez-Caballero^26,27^; María J. Fernandez-Nestosa^120^; Ramón Fernández^43,121^; Silvia Fernández Ferrero^11^; Yolanda Fernández Martínez^11^; Ana Fernández-Cruz^122^; Uxía Fernández-Robelo^123^; Amanda Fernández-Rodríguez^67,7^; Marta Fernández-Sampedro^43,45,44^; Ruth Fernández-Sánchez^26,27^; Tania Fernández-Villa^124^; Carmen Fernéndez Capitán^90^; Patricia Flores-Pérez^125^; Vicente Friaza^76,77^; Lácides Fuenmayor-Hernández^20^; Marta Fuertes Núñez^11^; Victoria Fumadó^126^; Ignacio Gadea^127^; Lidia Gagliardi^33,34^; Manuela Gago-Domínguez^128,101^; Natalia Gallego^129^; Cristina Galoppo^130^; Carlos Garcia-Cerrada^1,2,131^; Josefina Garcia-García^53^; Inés García^26,27^; Mercedes García^33,34^; Leticia García^33,34^; María Carmen García Torrejón^132,2^; Irene García-García^63^; Carmen García-Ibarbia^43,45,44^; Andrés C. García-Montero^133^; Ana García-Soidán^134^; Elisa García-Vázquez^53^; Aitor García-de-Vicuña^31,135^; Emiliano Garza-Frias^80^; Angela Gentile^130^; Belén Gil-Fournier^136^; Fernan Gonzalez Bernaldo de Quirós^137^; Manuel Gonzalez-Sagrado^92^; Hugo Gonzalo Benito^138^; Beatriz González Álvarez^107,108^; Anna González-Neira^28^; Javier González-Peñas^29,6,35^; Oscar Gorgojo-Galindo^139^; Florencia Guaragna^130^; Genilson P. Guegel^140^; Beatriz Guillen-Guio^96^; Encarna Guillen-Navarro^53,141,142,27^; Pablo Guisado-Vasco^113^; Luz D. Gutierrez-Castañeda^143,37^; Juan F. Gutiérrez-Bautista^144^; Luis Gómez Carrera^18^; María Gómez García^100^; Ángela Gómez Sacristán^145^; Javier Gómez-Arrue^107,108^; Mario Gómez-Duque^91,37^; Miguel Górgolas^72^; Sarah Heili-Frades^146^; Estefania Hernandez^147^; Luis D. Hernandez-Ortega^148,149^; Cristina Hernández Moro^11^; Guillermo Hernández-Pérez^79^; Rebeca Hernández-Vaquero^150^; Belen Herraez^28^; M. Teresa Herranz^53^; María Herrera^33,34^; María José Herrero^151,152^; Antonio Herrero-Gonzalez^153^; Juan P. Horcajada^154,155,14,156,7^; Natale Imaz-Ayo^31^; Maider Intxausti-Urrutibeaskoa^157^; Rafael H. Jacomo^158^; Rubén Jara^53^; Perez Maria Jazmin^130^; María A. Jimenez-Sousa^67,7^; Ángel Jiménez^33,34^; Pilar Jiménez^144^; Ignacio Jiménez-Alfaro^159^; Iolanda Jordan^160,161,76^; Rocío Laguna-Goya^162,163^; Daniel Laorden^18^; María Lasa-Lazaro^162,163^; María Claudia Lattig^78,164^; Ailen Lauriente^130^; Anabel Liger Borja^165^; Lucía Llanos^166^; Esther Lopez-Garcia^109,110,76,167^; Rosario Lopez-Rodriguez^26,27^; Leonardo Lorente^168^; José E. Lozano^169^; María Lozano-Espinosa^165^; Andre D. Luchessi^170^; Eduardo López Granados^171,172,27^; Amparo López-Bernús^79,56^; Miguel A. López-Ruz^173,174,175^; Ignacio Mahillo^176,177,97^; Esther Mancebo^162,163^; Carmen Mar^116^; Cristina Marcelo Calvo^90^; Miguel Marcos^79,56^; Alba Marcos-Delgado^178^; Pablo Mariscal Aguilar^18^; Marta Martin-Fernandez^179^; Laura Martin-Pedraza^60^; Amalia Martinez^180^; Iciar Martinez-Lopez^181,182^; Oscar Martinez-Nieto^49,164^; Pedro Martinez-Paz^138^; Angel Martinez-Perez^183^; Michel F. Martinez-Resendez^80^; María M. Martín^184^; María Dolores Martín^185^; Vicente Martín^178,76^; Caridad Martín-López^165^; José-Ángel Martín-Oterino^79,56^; María Martín-Vicente^67^; Ricardo Martínez^147^; Juan José Martínez^86,27^; Silvia Martínez^43,45^; Violeta Martínez Robles^11^; Eleno Martínez-Aquino^186^; Óscar Martínez-González^187^; Andrea Martínez-Ramas^26,27^; Laura Marzal^26,27^; Alicia Marín Candon^63^; Juliana F. Mazzeu^188,189,190^; Jeane F.P. Medeiros^38^; Kelliane A. Medeiros^191,192^; Francisco J. Medrano^75,76,77^; Xose M. Meijome^193,194^; Natalia Mejuto-Montero^195^; Humberto Mendoza Charris^64,20^; Eleuterio Merayo Macías^196^; Fátima Mercadillo^197^; Arieh R. Mercado-Sesma^148,149^; Pablo Minguez^26,27^; Antonio J J. Molina^178,76^; Elena Molina-Roldán^198^; Juan José Montoya^147^; Vitor M.S. Moraes^82^; Patricia Moreira-Escriche^199^; Xenia Morelos-Arnedo^64,20^; Victor Moreno Cuerda^1,2^; Alberto Moreno Fernández^90^; Antonio Moreno-Docón^53^; Junior Moreno-Escalante^20^; Rubén Morilla^77,200^; Patricia Muñoz García^201,97,6^; Ana Méndez-Echevarria^202^; Pablo Neira^130^; Julian Nevado^27,129,203^; Israel Nieto-Gañán^134^; Joana F.R. Nunes^40^; Rocio Nuñez- Torres^28^; Antònia Obrador-Hevia^204,205^; J. Gonzalo Ocejo-Vinyals^43,45^; Virginia Olivar^130^; Silviene F. Oliveira^188,206,207,208,209^; Lorena Ondo^26,27^; Alberto Orfao^22,23,24,25^; Luis Ortega^210^; Eva Ortega-Paino^48^; Fernando Ortiz-Flores^43,45^; Rocio Ortiz-Lopez^211,80^; José A. Oteo^12,212^; Harry Pachajoa^213,214^; Manuel Pacheco^147^; Fredy Javier Pacheco-Miranda^20^; Irene Padilla Conejo^11^; Sonia Panadero-Fajardo^83^; Mara Parellada^29,35,6^; Roberto Pariente-Rodríguez^134^; Estela Paz-Artal^162,163,215^; Germán Peces-Barba^216,97^; Miguel S. Pedromingo Kus^217^; Celia Perales^127^; Patricia Perez^218^; Gustavo Perez-de-Nanclares^31,219^; Teresa Perucho^220^; Lisbeth A. Pichardo^11^; Susana M.T. Pinho^191,221,222^; Mel·lina Pinsach-Abuin^68,66^; Luz Adriana Pinzón^91,37^; Guillermo Pita^28^; Francesc Pla-Junca^223,27^; Laura Planas-Serra^86,27^; Ericka N. Pompa-Mera^224^; Gloria L. Porras-Hurtado^147^; Aurora Pujol^86,27,225^; César Pérez^226^; Felipe Pérez-García^227,228^; Patricia Pérez-Matute^212^; Alexandra Pérez-Serra^68,66^; M. Elena Pérez-Tomás^53^; María Eugenia Quevedo Chávez^19,20^; Maria Angeles Quijada^30,229^; Inés Quintela^100^; Diana Ramirez-Montaño^230^; Soraya Ramiro León^136^; Pedro Rascado Sedes^231^; Delia Recalde^107,108^; Emma Recio-Fernández^212^; Salvador Resino^67,7^; Adriana P. Ribeiro^191,192,222^; Carlos S. Rivadeneira-Chamorro^37^; Diana Roa-Agudelo^49^; Montserrat Robelo Pardo^231^; Marilyn Johanna Rodriguez^37^; German Ezequiel Rodriguez Novoa^130^; Fernando Rodriguez-Artalejo^109,110,76,167^; Carlos Rodriguez-Gallego^232,233^; José A. Rodriguez-Garcia^11^; María A. Rodriguez-Hernandez^71^; Antonio Rodriguez-Nicolas^144^; Agustí Rodriguez-Palmero^234,86^; Paula A. Rodriguez-Urrego^49^; Belén Rodríguez Maya^1^; Marena Rodríguez-Ferrer^20^; Emilio Rodríguez-Ruiz^231,101^; Federico Rojo^235,25^; Andrea Romero-Coronado^20^; Filomeno Rondón García^11^; Lidia S. Rosa^236^; Antonio Rosales-Castillo^237^; Cladelis Rubio^238,239^; María Rubio Olivera^33,34^; Montserrat Ruiz^86,27^; Francisco Ruiz-Cabello^144,174,240^; Eva Ruiz-Casares^220^; Juan J. Ruiz-Cubillan^43,45^; Javier Ruiz-Hornillos^241,34,242^; Pablo Ryan^243,244,245^; Hector D. Salamanca^36,37^; Lorena Salazar-García^78^; Giorgina Gabriela Salgueiro Origlia ^90^; Cristina Sancho- Sainz^157^; Anna Sangil^62^; Arnoldo Santos^226^; Ney P.C. Santos^118^; Agatha Schlüter^86,27^; Sonia Segovia^223,246,247^; Alex Serra-Llovich^248^; Fernando Sevil Puras^8^; Marta Sevilla Porras^27,129^; Miguel A. Sicolo^249,250^; Vivian N. Silbiger^170^; Nayara S. Silva^251^; Fabiola T.C. Silva^40^; Cristina Silván Fuentes^27^; Jordi Solé-Violán^252,97,253^; José Manuel Soria^183^; Jose V. Sorlí^94,95^; Renata R. Sousa^188^; Juan Carlos Souto^3^; Karla S.C. Souza^84^; Vanessa S. Souza^104^; John J. Sprockel^91,37^; David A. Suarez-Zamora^49^; José Javier Suárez-Rama^100^; Pedro-Luis Sánchez^114,56^; Antonio J. Sánchez López^254^; María Concepción Sánchez Prados^18^; Javier Sánchez Real^11^; Jorge Sánchez Redondo^1,255^; Clara Sánchez-Pablo^114^; Olga Sánchez-Pernaute^256^; Xiana Taboada-Fraga^195^; Eduardo Tamayo^257,139^; Alvaro Tamayo-Velasco^258^; Juan Carlos Taracido-Fernandez^153^; Nathali A.C. Tavares^259^; Carlos Tellería^107,108^; Jair Antonio Tenorio Castaño^27,129,203^; Alejandro Teper^130^; Ronald P. Torres Gutiérrez^217^; Juan Torres-Macho^260^; Lilian Torres-Tobar^37^; Jesús Troya^243^; Miguel Urioste^197^; Juan Valencia-Ramos^261^; Agustín Valido^21,262^; Juan Pablo Vargas Gallo^263,264^; Belén Varón^265^; Romero H.T. Vasconcelos^259^; Tomas Vega^266^; Santiago Velasco-Quirce^267^; Julia Vidán Estévez^11^; Miriam Vieitez-Santiago^43,45^; Carlos Vilches^268^; Lavinia Villalobos^11^; Felipe Villar^216^; Judit Villar-Garcia^269,270,271^; Cristina Villaverde^26,27^; Pablo Villoslada-Blanco^212^; Ana Virseda-Berdices^67^; Valentina Vélez-Santamaría^85,86^; Virginia Víctor^33,34^; Zuleima Yáñez^20^; Antonio Zapatero-Gaviria^272^; Ruth Zarate^273^; Sandra Zazo^235^; Gabriela V. da Silva^39^; Raimundo de Andrés^274^; Jéssica N.G. de Araújo^251^; Carmen de Juan^199^; Julianna Lys de Sousa Alves Neri^275^; Carmen de la Horra^77^; Ana B. de la Hoz^31^; Victor del Campo-Pérez^276^; Manoella do Monte Alves^277,278^; Katiusse A. dos Santos^84^; Yady Álvarez-Benítez^19,20^; Felipe Álvarez-Navia^79,56^; María Íñiguez^212^; Miguel López de Heredia^27^; Ingrid Mendes^27^; Rocío Moreno^27^; Esther Sande^27,101,102^; Carlos Flores^279,96,97,233^; José A. Riancho^43,44,45^; Augusto Rojas-Martinez^80^; Pablo Lapunzina^27,129,203^; Angel Carracedo^100,27,101,102,128^

### Scourge Cohort Group’s filiations (436 members)

^1^, Hospital Universitario Mostoles, Medicina Interna, Madrid, Spain

^2^, Universidad Francisco de Vitoria, Madrid, Spain

^3^, Haemostasis and Thrombosis Unit, Hospital de la Santa Creu i Sant Pau, IIB Sant Pau, Barcelona, Spain

^4^, Unit of Infectious Diseases, Hospital Universitario 12 de Octubre, Instituto de Investigación Sanitaria Hospital 12 de Octubre (imas12), Madrid, Spain

^5^, Spanish Network for Research in Infectious Diseases (REIPI RD16/0016/0002), Instituto de Salud Carlos III, Madrid, Spain

^6^, School of Medicine, Universidad Complutense, Madrid, Spain

^7^, Centro de Investigación Biomédica en Red de Enfermedades Infecciosas (CIBERINFEC), Instituto de Salud Carlos III, Madrid, Spain

^8^, Hospital General Santa Bárbara de Soria, Soria, Spain

^9^, Pediatric Neurology Unit, Department of Pediatrics, Navarra Health Service Hospital, Pamplona, Spain

^10^, Navarra Health Service, NavarraBioMed Research Group, Pamplona, Spain

^11^, Complejo Asistencial Universitario de León, León, Spain

^12^, Hospital Universitario San Pedro, Infectious Diseases Department, Logroño, Spain

^13^, Fundación Institut Guttmann, Institut Universitari de Neurorehabilitació adscrit a la UAB, Hospital de Neurorehabilitació, Barcelona, Spain

^14^, Universitat Autònoma de Barcelona (UAB), Barcelona, Spain

^15^, Fundació Institut d’Investigació en Ciències de la Salut Germans Trias i Pujol, Barcelona, Spain

^16^, Hospital General de Occidente, Guadalajara, Mexico

^17^, Microbiology Unit, Hospital Universitario N. S. de Candelaria, Santa Cruz de Tenerife, Spain

^18^, Hospital Universitario La Paz-IDIPAZ, Servicio de Neumología, Madrid, Spain

^19^, Camino Universitario Adelita de Char, Mired IPS, Barranquilla, Colombia

^20^, Universidad Simón Bolívar, Facultad de Ciencias de la Salud, Barranquilla, Colombia

^21^, Hospital Universitario Virgen Macarena, Neumología, Seville, Spain

^22^, Departamento de Medicina, Universidad de Salamanca, Salamanca, Spain

^23^, Centro de Investigación del Cáncer (IBMCC) Universidad de Salamanca - CSIC, Salamanca, Spain

^24^, Biomedical Research Institute of Salamanca (IBSAL) Salamanca, Spain

^25^, Centre for Biomedical Network Research on Cancer (CIBERONC), Instituto de Salud Carlos III, Madrid, Spain

^26^, Department of Genetics & Genomics, Instituto de Investigación Sanitaria-Fundación Jiménez Díaz University Hospital - Universidad Autónoma de Madrid (IIS-FJD, UAM), Madrid, Spain

^27^, Centre for Biomedical Network Research on Rare Diseases (CIBERER), Instituto de Salud Carlos III, Madrid, Spain

^28^, Spanish National Cancer Research Centre, Human Genotyping-CEGEN Unit, Madrid, Spain

^29^, Department of Child and Adolescent Psychiatry, Institute of Psychiatry and Mental Health, Hospital General Universitario Gregorio Marañón (IiSGM), Madrid, Spain

^30^, Clinical Pharmacology Service, Hospital de la Santa Creu i Sant Pau, IIB Sant Pau, Barcelona, Spain

^31^, Biocruces Bizkai HRI, Barakaldo, Bizkaia, Spain

^32^, Cruces University Hospital, Osakidetza, Barakaldo, Bizkaia, Spain

^33^, Hospital Infanta Elena, Valdemoro, Madrid, Spain

^34^, Instituto de Investigación Sanitaria-Fundación Jiménez Díaz University Hospital - Universidad Autónoma de Madrid (IIS-FJD, UAM), Madrid, Spain

^35^, Centre for Biomedical Network Research on Mental Health (CIBERSAM), Instituto de Salud Carlos III, Madrid, Spain

^36^, Fundación Hospital Infantil Universitario de San José, Bogotá, Colombia

^37^, Fundación Universitaria de Ciencias de la Salud, Bogotá, Colombia

^38^, Universidade Federal do Rio Grande do Norte, Programa de Pós-graduação em Ciências da Saúde, Natal, Brazil

^39^, Universidade Federal do Rio Grande do Norte, Departamento de Medicina Clínica, Natal, Brazil

^40^, Departamento de Genética e Morfologia, Instituto de Ciências Biológicas, Universidade de Brasília, Brasilia, Brazil

^41^, Colégio Marista de Brasilia, Brazil

^42^, Associação Brasileira de Educação e Cultura, Brazil

^43^, IDIVAL, Santander, Spain

^44^, Universidad de Cantabria, Santander, Spain

^45^, Hospital U M Valdecilla, Santander, Spain

^46^, Hospital Universitario La Paz-IDIPAZ, Servicio de Medicina Interna, Madrid, Spain

^47^, Fundació Docència I Recerca Mutua Terrassa, Barcelona, Spain

^48^, Spanish National Cancer Research Center, CNIO Biobank, Madrid, Spain

^49^, Fundación Santa Fe de Bogota, Departamento Patologia y Laboratorios, Bogotá, Colombia

^50^, Hospital General de Occidente, Zapopan, Jalisco, Mexico

^51^, Centro Universitario de Tonalá, Universidad de Guadalajara, Tonalá, Jalisco, Mexico

^52^, Centro de Investigación Multidisciplinario en Salud, Universidad de Guadalajara, Tonalá, Jalisco, Mexico

^53^, Instituto Murciano de Investigación Biosanitaria (IMIB-Arrixaca), Murcia, Spain

^54^, Universidad Católica San Antonio de Murcia (UCAM), Murcia, Spain

^55^, Hospital Universitario de Salamanca-IBSAL, Servicio de Medicina Interna-Unidad de Enfermedades Infecciosas, Salamanca, Spain

^56^, Universidad de Salamanca, Salamanca, Spain

^57^, Hospital Universitario de Fuenlabrada, Department of Internal Medicine, Madrid, Spain

^58^, Escola Tecnica de Saúde, Laboratorio de Vigilancia Molecular Aplicada, Pará, Brazil

^59^, Federal University of Pernambuco, Genetics Postgraduate Program, Recife, PE, Brazil

^60^, Hospital Universitario Infanta Leonor, Servicio de Alergia, Madrid, Spain

^61^, Hospital Universitario del Tajo, Servicio de Medicina Intensiva, Aranjuez, Spain

^62^, Hospital Universitario Mutua Terrassa, Barcelona, Spain

^63^, Hospital Universitario La Paz-IDIPAZ, Servicio de Farmacología, Madrid, Spain

^64^, Alcaldía de Barranquilla, Secretaría de Salud, Barranquilla, Colombia

^65^, Instituto de Investigación Sanitaria de Santiago (IDIS), Xenética Cardiovascular, Santiago de Compostela, Spain

^66^, Centre for Biomedical Network Research on Cardiovascular Diseases (CIBERCV), Instituto de Salud Carlos III, Madrid, Spain

^67^, Unidad de Infección Viral e Inmunidad, Centro Nacional de Microbiología (CNM), Instituto de Salud Carlos III (ISCIII), Madrid, Spain

^68^, Cardiovascular Genetics Center, Institut d’Investigació Biomèdica Girona (IDIBGI), Girona, Spain

^69^, Medical Science Department, School of Medicine, University of Girona, Girona, Spain

^70^, Hospital Josep Trueta, Cardiology Service, Girona, Spain

^71^, Institute of Biomedicine of Seville (IBiS), Consejo Superior de Investigaciones Científicas (CSIC)- University of Seville- Virgen del Rocio University Hospital, Seville, Spain

^72^, Division of Infectious Diseases, Instituto de Investigación Sanitaria-Fundación Jiménez Díaz University Hospital - Universidad Autónoma de Madrid (IIS-FJD, UAM), Madrid, Spain

^73^, Intensive Care Unit, Hospital Universitario Insular de Gran Canaria, Las Palmas de Gran Canaria, Spain

^74^, Hospital Universitario Mutua Terrassa, Terrassa, Spain

^75^, Departemento de Medicina, Hospital Universitario Virgen del Rocío,Universidad de Sevilla, Seville, Spain

^76^, Centre for Biomedical Network Research on Epidemiology and Public Health (CIBERESP), Instituto de Salud Carlos III, Madrid, Spain

^77^, Instituto de Biomedicina de Sevilla, Seville, Spain

^78^, Universidad de los Andes, Facultad de Ciencias, Bogotá, Colombia

^79^, Hospital Universitario de Salamanca-IBSAL, Servicio de Medicina Interna, Salamanca, Spain

^80^, Tecnologico de Monterrey, Escuela de Medicina y Ciencias de la Salud and Hospital San Jose TecSalud, Monterrey, Mexico

^81^, University of Fortaleza (UNIFOR), Department of Nutrition. Fortaleza, Brazil

^82^, Departamento de Química, Faculdade de Filosofia, Ciências e Letras de Ribeirão Preto, Universidade de São Paulo, Brazil

^83^, Andalusian Public Health System Biobank, Granada, Spain

^84^, Universidade Federal do Rio Grande do Norte, Programa de Pós-Graduação em Ciências Farmacêuticas, Natal, Brazil

^85^, Neuromuscular Unit, Neurology Department, Hospital Universitari de Bellvitge, L’Hospitalet de Llobregat (Barcelona), Spain

^86^, Bellvitge Biomedical Research Institute (IDIBELL), Neurometabolic Diseases Laboratory, L’Hospitalet de Llobregat, Spain

^87^, Centre for Biomedical Network Research on Diabetes and Metabolic Associated Diseases (CIBERDEM), Instituto de Salud Carlos III, Madrid, Spain

^88^, University of Pais Vasco, UPV/EHU, Bizkaia, Spain

^89^, Oncology and Genetics Unit, Instituto de Investigacion Sanitaria Galicia Sur, Xerencia de Xestion Integrada de Vigo-Servizo Galego de Saúde, Vigo, Spain

^90^, Hospital Universitario La Paz, Hospital Carlos III, Madrid, Spain

^91^, Hospital de San José, Sociedad de Cirugía de Bogota, Bogotá, Colombia

^92^, Hospital Universitario Río Hortega, Valladolid, Spain

^93^, Servicio de Medicina intensiva, Complejo Hospitalario Universitario de A Coruña (CHUAC), Sistema Galego de Saúde (SERGAS), A Coruña, Spain

^94^, Valencia University, Preventive Medicine Department, Valencia, Spain

^95^, Centre for Biomedical Network Research on Physiopatology of Obesity and Nutrition (CIBEROBN), Instituto de Salud Carlos III, Madrid, Spain

^96^, Research Unit, Hospital Universitario N.S. de Candelaria, Santa Cruz de Tenerife, Spain

^97^, Centre for Biomedical Network Research on Respiratory Diseases (CIBERES), Instituto de Salud Carlos III, Madrid, Spain

^98^, Otto von Guericke University, Departament of Microgravity and Translational Regenerative Medicine, Magdeburg, Germany

^99^, Maternidade Escola Janário Cicco, Natal, Brazil

^100^, Centro Nacional de Genotipado (CEGEN), Universidade de Santiago de Compostela, Santiago de Compostela, Spain

^101^, Instituto de Investigación Sanitaria de Santiago (IDIS), Santiago de Compostela, Spain

^102^, Centro Singular de Investigación en Medicina Molecular y Enfermedades Crónicas (CIMUS), Universidade de Santiago de Compostela, Santiago de Compostela, Spain

^103^, Institute of Psychiatry and Mental Health, Hospital General Universitario Gregorio Marañón (IiSGM), Madrid, Spain

^104^, Programa de Pós Graduação em Ciências da Saúde, Faculdade de Medicina, Universidade de Brasília, Brasilia, Brazil

^105^, Fundació Docència I Recerca Mutua Terrassa, Terrassa, Spain

^106^, Hospital Universitario Mostoles, Unidad de Genética, Madrid, Spain

^107^, Instituto Aragonés de Ciencias de la Salud (IACS), Zaragoza, Spain

^108^, Instituto Investigación Sanitaria Aragón (IIS-Aragon), Zaragoza, Spain

^109^, Department of Preventive Medicine and Public Health, School of Medicine, Universidad Autónoma de Madrid, Madrid, Spain

^110^, IdiPaz (Instituto de Investigación Sanitaria Hospital Universitario La Paz), Madrid, Spain

^111^, Hospital Universitario Virgen del Rocío, Servicio de Medicina Interna, Seville, Spain

^112^, Unidad Diagnóstico Molecular. Fundación Rioja Salud, La Rioja, Spain

^113^, Hospital Universitario Quironsalud Madrid, Madrid, Spain

^114^, Hospital Universitario de Salamanca-IBSAL, Servicio de Cardiología, Salamanca, Spain

^115^, Hospital Universitario Puerta de Hierro, Servicio de Medicina Interna, Majadahonda, Spain

^116^, Biocruces Bizkaia Health Research Institute, Galdakao University Hospital, Osakidetza, Bizkaia, Spain

^117^, Instituto Regional de Investigación en Salud-Universidad Nacional de Caaguazú, Caaguazú, Paraguay

^118^, Universidade Federal do Pará, Núcleo de Pesquisas em Oncologia, Belém, Pará, Brazil

^119^, Hospital Ophir Loyola, Departamento de Ensino e Pesquisa, Belém, Pará, Brazil

^120^, Universidad Nacional de Asunción, Facultad de Politécnica, Paraguay

^121^, Fundación Asilo San Jose, Santander, Spain

^122^, Unidad de Enfermedades Infecciosas, Servicio de Medicina Interna, Hospital Universitario Puerta de Hierro, Instituto de Investigación Sanitaria Puerta de Hierro - Segovia de Arana, Madrid, Spain

^123^, Urgencias Hospitalarias, Complejo Hospitalario Universitario de A Coruña (CHUAC), Sistema Galego de Saúde (SERGAS), A Coruña, Spain

^124^, Grupo de Investigación en Interacciones Gen-Ambiente y Salud (GIIGAS) - Instituto de Biomedicina (IBIOMED), Universidad de León, León, Spain

^125^, Hospital Universitario Niño Jesús, Pediatrics Department, Madrid, Spain

^126^, Unitat de Malalties Infeccioses i Importades, Servei de Pediatría, Infectious and Imported Diseases, Pediatric Unit, Hospital Universitari Sant Joan de Deú, Barcelona, Spain

^127^, Microbiology Department, Instituto de Investigación Sanitaria-Fundación Jiménez Díaz University Hospital - Universidad Autónoma de Madrid (IIS-FJD, UAM), Madrid, Spain

^128^, Fundación Pública Galega de Medicina Xenómica, Sistema Galego de Saúde (SERGAS) Santiago de Compostela, Spain

^129^, Instituto de Genética Médica y Molecular (INGEMM), Hospital Universitario La Paz-IDIPAZ, Madrid, Spain

^130^, Hospital de Niños Ricardo Gutierrez, Buenos Aires, Argentina

^131^, Centre for Biomedical Network Research on Rare Diseases (CIBERER), Instituto de Salud Carlos III, Madrid, Spain Universidad Francisco de Vitoria, Madrid,Spain

^132^, Hospital Infanta Elena, Servicio de Medicina Intensiva, Valdemoro, Madrid, Spain

^133^, University of Salamanca, Biomedical Research Institute of Salamanca (IBSAL), Salamanca, Spain

^134^, Department of Immunology, IRYCIS, Hospital Universitario Ramón y Cajal, Madrid, Spain

^135^, Osakidetza, Cruces University Hospital, Bizkaia, Spain

^136^, Hospital Universitario de Getafe, Servicio de Genética, Madrid, Spain

^137^, Ministerio de Salud Ciudad de Buenos Aires, Buenos Aires, Argentina

^138^, Hospital Clinico Universitario de Valladolid, Unidad de Apoyo a la Investigación, Valladolid, Spain

^139^, Universidad de Valladolid, Departamento de Cirugía, Valladolid, Spain

^140^, Secretaria Municipal de Saude de Apodi, Natal, Brazil

^141^, Sección Genética Médica - Servicio de Pediatría, Hospital Clínico Universitario Virgen de la Arrixaca, Servicio Murciano de Salud, Murcia, Spain

^142^, Departamento Cirugía, Pediatría, Obstetricia y Ginecología, Facultad de Medicina, Universidad de Murcia (UMU), Murcia, Spain

^143^, Hospital Universitario Centro Dermatológico Federico Lleras Acosta, Bogotá, Colombia

^144^, Hospital Universitario Virgen de las Nieves, Servicio de Análisis Clínicos e Inmunología, Granada, Spain

^145^, Pneumology Department, Hospital General Universitario Gregorio Marañón (iiSGM), Madrid, Spain

^146^, Intermediate Respiratory Care Unit, Department of Pneumology, Instituto de Investigación Sanitaria-Fundación Jiménez Díaz University Hospital - Universidad Autónoma de Madrid (IIS-FJD, UAM), Madrid, Spain

^147^, Clinica Comfamiliar Risaralda, Pereira, Colombia

^148^, Centro Universitario de Tonalá, Universidad de Guadalajara, Guadalajara, Mexico

^149^, Centro de Investigación Multidisciplinario en Salud, Universidad de Guadalajara, Guadalajara, Mexico

^150^, Unidad de Cuidados, Intensivos Hospital Clínico Universitario de Santiago (CHUS), Sistema Galego de Saúde (SERGAS), Santiago de Compostela, Spain

^151^, IIS La Fe, Plataforma de Farmacogenética, Valencia, Spain

^152^, Universidad de Valencia, Departamento de Farmacología, Valencia, Spain

^153^, Data Analysis Department, Instituto de Investigación Sanitaria-Fundación Jiménez Díaz University Hospital - Universidad Autónoma de Madrid (IIS-FJD, UAM), Madrid, Spain

^154^, Hospital del Mar, Infectious Diseases Service, Barcelona, Spain

^155^, Institut Hospital del Mar d’Investigacions Mèdiques (IMIM), Barcelona, Spain

^156^, CEXS-Universitat Pompeu Fabra, Spanish Network for Research in Infectious Diseases (REIPI), Barcelona, Spain

^157^, Biocruces Bizkaia Health Research Institute, Basurto University Hospital, Osakidetza, Bizkaia, Spain

^158^, Sabin Medicina Diagnóstica, Brazil

^159^, Opthalmology Department, Instituto de Investigación Sanitaria-Fundación Jiménez Díaz University Hospital - Universidad Autónoma de Madrid (IIS-FJD, UAM), Madrid, Spain

^160^, Hospital Sant Joan de Deu,Pediatric Critical Care Unit, Barcelona, Spain

^161^, Paediatric Intensive Care Unit, Agrupación Hospitalaria Clínic-Sant Joan de Déu, Esplugues de Llobregat, Barcelona, Spain

^162^, Hospital Universitario 12 de Octubre, Department of Immunology, Madrid, Spain

^163^, Instituto de Investigación Sanitaria Hospital 12 de Octubre (imas12), Transplant Immunology and Immunodeficiencies Group, Madrid, Spain

^164^, SIGEN Alianza Universidad de los Andes - Fundación Santa Fe de Bogotá, Bogotá, Colombia

^165^, Hospital General de Segovia, Medicina Intensiva, Segovia, Spain

^166^, Clinical Trials Unit, Instituto de Investigación Sanitaria-Fundación Jiménez Díaz University Hospital - Universidad Autónoma de Madrid (IIS-FJD, UAM), Madrid, Spain

^167^, IMDEA-Food Institute, CEI UAM+CSIC, Madrid, Spain

^168^, Intensive Care Unit, Hospital Universitario de Canarias, La Laguna, Spain

^169^, Dirección General de Salud Pública, Consejería de Sanidad, Junta de Castilla y León, Valladolid, Spain

^170^, Universidade Federal do Rio Grande do Norte, Departamento de Analises Clinicas e Toxicologicas, Natal, Brazil

^171^, Hospital Universitario La Paz-IDIPAZ, Servicio de Inmunología, Madrid, Spain

^172^, La Paz Institute for Health Research (IdiPAZ), Lymphocyte Pathophysiology in Immunodeficiencies Group, Madrid, Spain

^173^, Hospital Universitario Virgen de las Nieves, Servicio de Enfermedades Infecciosas, Granada, Spain

^174^, Instituto de Investigación Biosanitaria de Granada (ibs GRANADA), Granada, Spain

^175^, Universidad de Granada, Departamento de Medicina, Granada, Spain

^176^, Fundación Jiménez Díaz, Epidemiology, Madrid, Spain

^177^, Universidad Autónoma de Madrid, Department of Medicine, Madrid, Spain

^178^, Instituto de Biomedicina (IBIOMED), Universidad de León, León, Spain

^179^, Universidad de Valladolid, Departamento de Medicina, Valladolid, Spain

^180^, Hospital Universitario Infanta Leonor, Servicio de Medicina Intensiva, Madrid, Spain

^181^, Unidad de Genética y Genómica Islas Baleares, Islas Baleares, Spain

^182^, Hospital Universitario Son Espases, Unidad de Diagnóstico Molecular y Genética Clínica, Islas Baleares, Spain

^183^, Genomics of Complex Diseases Unit, Research Institute of Hospital de la Santa Creu i Sant Pau, IIB Sant Pau, Barcelona, Spain

^184^, Intensive Care Unit, Hospital Universitario N. S. de Candelaria, Santa Cruz de Tenerife, Spain

^185^, Preventive Medicine Department, Instituto de Investigación Sanitaria-Fundación Jiménez Díaz University Hospital - Universidad Autónoma de Madrid (IIS-FJD, UAM), Madrid, Spain

^186^, Servicio de Medicina Interna, Sanatorio Franchin, Buenos Aires, Argentina

^187^, Hospital Universitario del Tajo, Servicio de Medicina Intensiva, Toledo, Spain

^188^, Faculdade de Medicina, Universidade de Brasília, Brasilia, Brazil

^189^, Programa de Pós-Graduação em Ciências Médicas, Universidade de Brasília, Brasilia, Brazil

^190^, Programa de Pós-Graduação em Ciências da Saúde, Universidade de Brasília, Brasilia, Brazil

^191^, Hospital das Forças Armadas, Brazil

^192^, Exército Brasileiro, Brazil

^193^, Hospital El Bierzo, Gerencia de Asistencia Sanitaria del Bierzo (GASBI), Gerencia Regional de Salud (SACYL), Ponferrada, Spain

^194^, Grupo INVESTEN, Instituto de Salud Carlos III, Madrid, Spain

^195^, Unidad de Cuidados Intensivos, Complejo Universitario de A Coruña (CHUAC), Sistema Galego de Saúde (SERGAS), A Coruña, Spain

^196^, Hospital El Bierzo, Unidad Cuidados Intensivos, León, Spain

^197^, Spanish National Cancer Research Centre, Familial Cancer Clinical Unit, Madrid, Spain

^198^, Instituto de Investigación Sanitaria San Carlos (IdISSC), Hospital Clínico San Carlos (HCSC), Madrid, Spain

^199^, Hospital Universitario Severo Ochoa, Servicio de Medicina Interna, Madrid, Spain

^200^, Universidad de Sevilla, Departamento de Enfermería, Seville, Spain

^201^, Hospital General Universitario Gregorio Marañón (IiSGM), Madrid, Spain

^202^, Hospital Universitario La Paz-IDIPAZ, Servicio de Pediatría, Madrid, Spain

^203^, ERN-ITHACA-European Reference Network

^204^, Unidad de Genética y Genómica Islas Baleares, Unidad de Diagnóstico Molecular y Genética Clínica, Hospital Universitario Son Espases, Islas Baleares, Spain

^205^, Instituto de Investigación Sanitaria Islas Baleares (IdISBa), Islas Baleares, Spain

^206^, Programa de Pós-Graduação em Biologia Animal, Universidade de Brasília, Brasília, Brazil

^207^, Programa de Pós-Graduação em Ciências da Saúde, Universidade de Brasília, Brasília, Brazil

^208^, Programa de Pós-Graduação Profissional em Ensino de Biologia, Universidade de Brasília, Brasília, Brazil

^209^, Programa de Pós-Graduação em Ciências Médicas, Universidade de Brasília, Brasília, Brazil

^210^, Anatomía Patológica, Instituto de Investigación Sanitaria San Carlos (IdISSC), Hospital Clínico San Carlos (HCSC), Madrid, Spain

^211^, Tecnológico de Monterrey, Monterrey, Mexico

^212^, Infectious Diseases, Microbiota and Metabolism Unit, Center for Biomedical Research of La Rioja (CIBIR), Logroño, Spain

^213^, Centro de Investigación en Anomalías Congénitas y Enfermedades Raras (CIACER), Universidad Icesi

^214^, Departamento de Genetica, Fundación Valle del Lili

^215^, Universidad Complutense de Madrid, Department of Immunology, Ophthalmology and ENT, Madrid, Spain

^216^, Department of Neumology, Instituto de Investigación Sanitaria-Fundación Jiménez Díaz University Hospital - Universidad Autónoma de Madrid (IIS-FJD, UAM), Madrid, Spain

^217^, Hospital Nuestra Señora de Sonsoles, Ávila, Spain

^218^, Inditex, A Coruña, Spain

^219^, Osakidetza, Cruces University Hospital, Barakaldo, Bizkaia, Spain

^220^, GENYCA, Madrid, Spain

^221^, Marinha do Brasil, Brazil

^222^, Universidade de Brasília, Brasilia, Brazil

^223^, Neuromuscular Diseases Unit, Department of Neurology, Hospital de la Santa Creu i Sant Pau, Universitat Autònoma de Barcelona, Barcelona, Spain

^224^, Instituto Mexicano del Seguro Social (IMSS), Centro Médico Nacional Siglo XXI, Unidad de Investigación Médica en Enfermedades Infecciosas y Parasitarias, Mexico City, Mexico

^225^, Catalan Institution of Research and Advanced Studies (ICREA), Barcelona, Spain

^226^, Intensive Care Department, Instituto de Investigación Sanitaria-Fundación Jiménez Díaz University Hospital - Universidad Autónoma de Madrid (IIS-FJD, UAM), Madrid, Spain

^227^, Hospital Universitario Príncipe de Asturias, Servicio de Microbiología Clínica, Madrid, Spain

^228^, Universidad de Alcalá de Henares, Departamento de Biomedicina y Biotecnología, Facultad de Medicina y Ciencias de la Salud, Madrid, Spain

^229^, Drug Research Centre, Institut d’Investigació Biomèdica Sant Pau, IIB-Sant Pau, Barcelona, Spain

^230^, Departamento de Genetica, Clinica imbanaco

^231^, Unidad de Cuidados Intensivos, Hospital Clínico Universitario de Santiago (CHUS), Sistema Galego de Saúde (SERGAS), Santiago de Compostela, Spain

^232^, Department of Immunology, Hospital Universitario de Gran Canaria Dr. Negrín, Las Palmas de Gran Canaria, Spain

^233^, Department of Clinical Sciences, University Fernando Pessoa Canarias, Las Palmas de Gran Canaria, Spain

^234^, University Hospital Germans Trias i Pujol, Pediatrics Department, Badalona, Spain

^235^, Department of Pathology, Biobank, Instituto de Investigación Sanitaria-Fundación Jiménez Díaz University Hospital - Universidad Autónoma de Madrid (IIS-FJD, UAM), Madrid, Spain

^236^, Faculdade de Ciências da Saúde, Universidade de Brasília, Brasilia, Brazil

^237^, Hospital Universitario Virgen de las Nieves, Servicio de Medicina Interna, Granada, Spain

^238^, Fundación Universitaria de Ciencias de la Salud, Grupo de Ciencias Básicas en Salud (CBS), Bogotá, Colombia

^239^, Sociedad de Cirugía de Bogotá, Hospital de San José, Bogotá, Colombia

^240^, Universidad de Granada, Departamento Bioquímica, Biología Molecular e Inmunología III, Granada, Spain

^241^, Hospital Infanta Elena, Allergy Unit, Valdemoro, Madrid, Spain

^242^, Faculty of Medicine, Universidad Francisco de Vitoria, Madrid, Spain

^243^, Hospital Universitario Infanta Leonor, Madrid, Spain

^244^, Complutense University of Madrid, Madrid, Spain

^245^, Gregorio Marañón Health Research Institute (IiSGM), Madrid, Spain

^246^, The John Walton Muscular Dystrophy Research Centre, Newcastle University and Newcastle Hospitals NHS Foundation Trust, Newcastle upon Tyne, UK.

^247^, Neuromuscular Unit, Neuropediatrics Department, Institut de Recerca Sant Joan de Déu, Hospital Sant Joan de Déu, Spain

^248^, Fundació Docència i Recerca Mutua Terrassa, Terrassa, Spain

^249^, Casa de Saúde São Lucas, Natal, Brazil

^250^, Hospital Rio Grande, Rio Grande do Norte, Natal, Brazil

^251^, Universidade Federal do Rio Grande do Norte, Pós-graduação em Biotecnologia - Rede de Biotecnologia do Nordeste (Renorbio), Natal, Brazil

^252^, Intensive Care Unit, Hospital Universitario de Gran Canaria Dr. Negrín, Las Palmas de Gran Canaria, Spain

^253^, Universidad Fernando Pessoa Canarias, Las Palmas de Gran Canaria, Spain

^254^, Biobank, Puerta de Hierro-Segovia de Arana Health Research Institute, Madrid, Spain

^255^, Universidad Rey Juan Carlos, Madrid, Spain

^256^, Reumathology Service, Instituto de Investigación Sanitaria-Fundación Jiménez Díaz University Hospital - Universidad Autónoma de Madrid (IIS-FJD, UAM), Madrid, Spain

^257^, Hospital Clinico Universitario de Valladolid, Servicio de Anestesiologia y Reanimación, Valladolid, Spain

^258^, Hospital Clinico Universitario de Valladolid, Servicio de Hematologia y Hemoterapia, Valladolid, Spain

^259^, Hospital Universitario Lauro Wanderley, Brazil

^260^, Hospital Universitario Infanta Leonor, Servicio de Medicina Interna, Madrid, Spain

^261^, University Hospital of Burgos, Burgos, Spain

^262^, Universidad de Sevilla, Seville, Spain

^263^, Fundación Santa Fe de Bogota, Instituto de servicios medicos de Emergencia y trauma, Bogotá, Colombia

^264^, Universidad de los Andes, Bogotá, Colombia

^265^, Quironprevención, A Coruña, Spain

^266^, Junta de Castilla y León, Consejería de Sanidad, Valladolid, Spain

^267^, Gerencia Atención Primaria de Burgos, Burgos, Spain

^268^, Immunogenetics-Histocompatibility group, Servicio de Inmunología, Instituto de Investigación Sanitaria Puerta de Hierro - Segovia de Arana, Madrid, Spain

^269^, Hospital del Mar, Department of Infectious Diseases, Barcelona, Spain

^270^, IMIM (Hospital del Mar Medical Research Institute, Institut Hospital del Mar d’Investigacions Mediques), Barcelona, Spain

^271^, Universitat Autònoma de Barcelona, Department of Medicine, Spain

^272^, Consejería de Sanidad, Comunidad de Madrid, Madrid, Spain

^273^, Centro para el Desarrollo de la Investigación Científica, Asunción, Paraguay

^274^, Internal Medicine Department, Instituto de Investigación Sanitaria-Fundación Jiménez Díaz University Hospital - Universidad Autónoma de Madrid (IIS-FJD, UAM), Madrid, Spain

^275^, Universidade Federal do Rio Grande do Norte, Programa de Pós Graduação em Nutrição, Natal, Brazil

^276^, Preventive Medicine Department, Instituto de Investigacion Sanitaria Galicia Sur, Xerencia de Xestion Integrada de Vigo-Servizo Galego de Saúde, Vigo, Spain

^277^, Universidade Federal do Rio Grande do Norte, Departamento de Infectologia, Natal, Brazil

^278^, Hospital de Doenças Infecciosas Giselda Trigueiro, Rio Grande do Norte, Natal, Brazil

^279^, Genomics Division, Instituto Tecnológico y de Energías Renovables, Santa Cruz de Tenerife, Spain
